# An empirical study to determine factors that motivate and limit the implementation Of ICT in healthcare environments

**DOI:** 10.1186/1472-6947-14-98

**Published:** 2014-12-23

**Authors:** Raj Gururajan, Abdul Hafeez-Baig

**Affiliations:** School of Management and Enterprise, University of Southern Queensland, West Street, Toowoomba, QLD 4350 Australia

**Keywords:** ICT, Healthcare, Information and Communication Technologies

## Abstract

**Background:**

The maturity and usage of wireless technology has influenced health services, and this has raised expectations from users that healthcare services will become more affordable due to technology growth. There is increasing evidence to justify this expectation, as telehealth is becoming more and more prevalent in many countries. Thus, health services are now offered beyond the boundaries of traditional hospitals, giving rise to many external factors dictating their quality. This has led us to investigate the factors that motivate and limit the implementation of ICT applications in the healthcare domain.

**Methods:**

We used a mixed method approach with the qualitative aspects leading the quantitative aspects. The main reason for this approach was to understand and explore the domain through the qualitative aspects as we could be part of the discussion. Then we conducted a quantitative survey to extract more responses in order to justify the claims explored in the qualitative process.

**Results:**

We found that there are a number of internal and external factors influencing ICT adoption in the healthcare environment so that services can be provided via ICT tools. These factors were grouped under factors contributing to improved outcomes, efficiency and the management of technology. We conceptualised that these three groups of factors drive ICT implementation to assure health services.

**Conclusions:**

The main lesson learned from this research was that Information Systems discipline needs to urgently consider health informatics as a serious growth area. We also found that as IS researchers, we need to ‘mix’ with the health environment in order to understand the environment and then develop suitable methods to answer posited research questions.

## Background

The Australian National Office of Information Economy has predicted that Australia is aligned to take advantage of the emerging information economy. It is true that Australia is among the leading countries in terms of internet infrastructure and

other technological developments, however, in comparison with other countries such as Finland, Canada, USA, and Sweden, the Australian healthcare service providers have been extremely slow to implement ICT technological developments such as wireless technology. Various contributing factors have been identified to explain the slow adoption of ICT technologies by researchers in this area, including a lack of management support, training and policies [1–4], the perceived lack of complexity and cost [5–9], the sensitive nature of information and the logistics involved in healthcare facilities [10–13], the nature and type of risk involved [9], the pressure for high quality of care, high litigation costs, and a lack of infrastructure and other resources [10]. Countries such as India and Pakistan have also caught up with advanced healthcare systems because of health tourism, and their systems are comparable to many western healthcare organisations in terms of ICT sophistication.

In spite of the slow rate of adoption of ICT technology in many healthcare systems, there is very little empirical research in this area [7]. Internationally, researchers have an increased interest in this area; however most of the research is dedicated to the technical and operational areas of ICT. There is very little empirical research into the factors that would lead to the successful adoption of ICT technologies in a given healthcare environment. Knowledge of critical success factors relating to the adoption of ICT technology will not only help to address other issues of adoption in the Australian healthcare system, it will also move forward research in this domain to develop a framework for such adoption.

Typically, background aspects of this study rely on earlier research into adoption, implementation, and innovation diffusion theories relevant to technology in general, including information systems, information technology, and computer technology. Wireless technology is not identical or analogous to any of these areas, and therefore any study which has concentrated on identifying factors or frameworks for the adoption of technology in general has limited applicability in the case of wireless technology for the healthcare system. Owing to the limitation on published results in this area, this study will be exploratory in nature. The long term objective of this study is aimed at developing a framework for the adoption of wireless technology in the Australian healthcare system, and identifying the relevant factors relating to this adoption.

As an starting point, we considered Rogers’ theory of innovation diffusion, as this is considered useful for understanding the facilitators and inhibitors of the implementation of technology in a given environment, because the theory provides an insight into the factors that influence the adoption of innovation.

Furthermore, Roger’s theory has been applied to many non ICT domains and so it is hoped that healthcare will also be a domain that has relevance to this theory.

Rogers’ theory [14] is primarily concerned with finding factors that influence the extent of adoption, and not the adoption process itself. Previous studies have defined three stages in the technology innovation cycle; adoption, implementation and post implementation [15–17]. Our study concentrates only on the adoption to implement ICT aspect, where the actual decision is made on ICT implementation in a healthcare facility. The decision to adopt depends purely on the drivers and inhibitors of the use of the chosen technology in a healthcare facility. It is anticipated, once the decision to implement the technology is taken, that the process of implementation will start. Once the technology has been implemented and used successfully, then the process of post-implementation will begin, in order to further understand the use of technology and the facilitation of its adoption. We followed this approach as many business process cycles follow the notion of planning, implementation and review, and the adoption, implementation and post-implementation model appears to suit business processes and workflow.

This study did not investigate the processes involved in the implementation of a technology, but assumed that a choice will be made to implement a technology in the healthcare facility, on the basis of identified business drivers. It is also anticipated that effective implementation will not take place at the time of delivery or installation of hardware or software applications; rather, it will happen over a time span dictated by drivers and inhibitors and supported by familiarity, knowledge base, policy framework, infrastructure, level of commitment, and trust, in order to be established and supported by various stakeholders. The main research aim is thus:

*What factors motivate and limit the implementation of ICT applications in a healthcare environment?*

### Research objectives

The specific objective of this research is to identify and determine factors that motivate and limit the implementation of ICT applications in the healthcare domain. This objective is formulated into the following two research questions:

What factors determine ICT developments and their implementation in a healthcare environment?

What factors limit the implementation of ICT developments in a healthcare environment?

### Scope of the study

This study is limited to healthcare facilities which have, or are in the process of implementing, innovative ICT developments. Even though it is understood that the implementation of ICT developments can vary from industry to industry, it is hoped that the findings of this research will have some impact on the adoption of ICT developments in other domains. It is also anticipated that this research will provide valuable insights into the current perception of ICT implementation and the factors that contribute towards such implementations in healthcare.

## Methods

It is anticipated that there will be a long list of variables that affect the adoption of wireless technology in the healthcare system. Therefore, in the absence of a comprehensive empirical study in this area, various factors have been identified and grouped through a systematic review of the available literature. The two broad groups of categories are internal factors and external factors; these two categories can further be subdivided into environmental factors, technological factors, organizational factors, and other mediating factors.

The two broader categories of internal and external factors are differentiated between the organizational-specific factors and non-organizational-specific factors, which are imposed on the organization by the external environment.

### Design

Morgan [18] mentions the use of qualitative approaches in social science research as a self-contained method, used as a supplementary source of data, or used in multi- method studies. While many techniques are available to capture data, in this study we employed multiple case studies, a focus group, and a survey technique to understand various issues influencing ICT usage in healthcare organisations. This combined approach was employed in order to elicit open-ended responses, to obtain factors that are not constrained by a pre-determined identification of constructs found in traditional surveys, and to determine the importance of such pre-determined factors [19, 20]. Furthermore, due to the exploratory nature of this study, this research is designed to capture a cross-sectional snapshot and a dynamic longitudinal picture of ICT usage in healthcare. Therefore, the research is carried out in in multiple phases.

### Multiple case studies

Multiple case studies were conducted to identify possible motivators for ICT implementation in healthcare organisations. Twenty private and public healthcare organisations were chosen in India, Pakistan and Australia with a total number of 80 staff interviewed in these organisations. We chose these organisations in these three countries as we are conducting funded research in these three countries and have connections in the healthcare sector. The focus of the interview was to explore the factors that motivate and limit ICT implementation. Hence, the unit of analysis is ‘organisational ICT factors or issues, including both internal and external factors’. The basic information of the interviewees is summarised in Tables 1 and 2. Table 1 indicates that the interviewees cover three main job levels: senior executives; middle managers; and operational staff. Table 2 summarises the seniority of interviewees. The percentage of interviewees who worked in the organisations for more than two years is over 90 per cent. This assisted the interviewers in better understanding the organisational environment and its working culture.

**Table 1:** **Descriptive analysis of the interviewers**

Job position of interviewee	Frequency	Percentage
Proprietors, partners, & executive	24	30.00%
Middle managers & professionals	39	48.75%
Operational staff	17	21.25%
Total	80	100%

**Table 2:** **Summary of demographics**

Seniority	Frequency	Percentage
2 years or under	5	6.25%
Over 2 and under 5 years	22	27.5%
Over 5 and under 10 years	16	20%
Over 10 years	36	45%
N/A	1	1.25%
Total	80	100%

Table 3 builds the linkages between the body of literature and the case studies. The enablers of organisational ICT implementation factors were identified from the interviews throughout the multiple case studies. We used text analysis software for extracting these enablers. The enablers mainly represent management aspects, and are congruent to business processes followed in the respective organisations. The results are shown in Table 3.

**Table 3:** **Summary of variables**

Enabler identified	Distributions (ranked)	Percentage
Learning	49	61.25%
Incentives & rewards	42	52.50%
Information technology infrastructure	19	23.75
T-Shaped skills	12	15.00%
Non-formalisation	11	13.75%
Mutual trust	9	11.25%
Non-centralisation	7	8.75%
Leadership	5	6.25%
Collaboration	3	3.75%

### Focus group interviews

Once the initial motivators (enablers) were identified, a focus group was conducted with an arbitrarily selected group to drill down the factors, with a view to understanding the ICT implementation aspects relating to these enablers. Seven individuals were selected from the 20 healthcare organisations, and these individuals were interviewed in order to explore their perception regarding the motivating and limiting factors of ICT implementation in their respective organisations. The selection was based on the size of the organisations, and involved healthcare organisations having at least 1000 beds. The group consisted of senior healthcare academics, clinical staff, health IT managers, and management practitioners. These individuals were chosen because they were involved, either directly or indirectly, with the ICT implementation in their organisations. Each interview was conducted over 2 hours, and the questions were open-ended. This provided direction to identify factors that influenced the ICT implementation. The primary objective of this exercise was to come up with an agreed upon, unique set of items under the heading of ‘factors influencing the implementation of ICT in healthcare environment’.

The set of motivating (Drivers) and limiting (Inhibitors) factors (as shown in Table 4) provided the scope for the study and a survey was administered based on these two sets of factors.

**Table 4:** **Summary of drivers and inhibitors**

Drivers	Inhibitors
Attract more practitioners	Administrative constraints
Better quality of service	Benefit evaluation barrier
Delivery of high quality information	Communication with colleagues
Easy access to data	Communication with physicians
Efficiency in communication	Complications in note taking due to difficult to read & write screens
Improved clinical flow	
Improved clinical performance	Device usage barrier
Improved delivery of information	Electronic medical records
Improved public image	Electronic prescribing
More contact time with patients	Legal barriers
Positive impact on patient safety	Patient education
Reduced inaccuracies	Problems in obtaining lab results
Reduced medical errors	Resource barrier
Reduced overall cost	
Saving effort	
Savings in time	
Reduced workload	

## Results

The results of this study pertain to stage 2, survey (evaluative). A survey instrument consisting of questions and multiple item scales was developed from the interview transcript. The main reason for this approach was that the initial set of participants (20 people) stated that previously tested instruments were inadequate for the purposes of this study. The data from interviews were used to develop a specific range of questions to gather a more detailed view from the wider population. The newly developed instrument was pilot-tested to capture the information reflecting the perceptions and practices of the industry, and particularly focused on what internal and external environmental factors shape the implementation of ICT and the extent of their influence. Prior to administering the survey, traditional validity checks such as face validity and peer review were performed. These checks were performed with people that have experience in questionnaire design methods.

The participants were chosen randomly from the internal telephone directory of the chosen organisations. The survey was then distributed to over 300 people in the chosen organisation. A total of 97 participants completed the survey. The reliability analysis returned a “Cronbach’s Alpha” value of 0.894, indicating a very high level of reliability [21]. Therefore the data collected from the survey was considered reliable and suitable for further statistical analysis. Furthermore, to be able to understand and identify the natural grouping of items from the questioners, an initial factor analysis was conducted on the data to identify factor groupings. An iterative process was employed to finally arrive at the following five factors. In deciding the factors, a loading value of 0.6 was set with varimax rotation. The groups were given appropriate titles in an arbitrary fashion based on the types of factors in each group. This final factor grouping is shown in the Table 5.

**Table 5:** **Summary of factor analysis**

Descriptions	Technology management	Data management	Improved outcome	Efficiency	Software limitation
Connection problems	.757				
Slow transfer rates	.756				
Interference with medical	.772				
Not able to access main	.817				
Not able to operate with	.779				
Frequent breakdown	.763				
Short battery life	.806				
Screen too small	.691				
Image not clear	.827				
Limited storage capacity	.665				
Not enough wireless	.811				
Not enough processing	.783				
Wireless device heavy to	.692				
Device stolen	.680				
Electronic medical record		.822			
Medical database referral		.834			
Electronic prescribing		.801			
Daily scheduling of		.794			
Obtain lab results		.845			
Billing and account		.802			
Disease state management		.766			
Administrative purposes		.815			
Generating “exceptions”		.771			
Patient education		.695			
Note taking		.720			
Drug administration		.642			
Communication with		.662			
Communication with		.678			
Enhance clinical			.878		
Attract more patients			.751		
High quality information			.774		
Easy access to data			.800		
Improve patient safety			.776		
Save time				.829	
Save effort				.759	
Enhance clinical flow				.784	
lack of solution					.748
Inadequate resources					.693
Migration issues					.684

To understand the relationship between variables identified through factor analysis and healthcare professionals’ views on ICT implementation factors, a multiple regression analysis technique was adopted. A multiple regression analysis was conducted though the “Enter Method” for the independent variables (Technology Management, Data Management, Improved Outcomes, Efficiency, and Software Limitations) and dependent variable “Intention to implement ICT” from the data collected. As can be seen from Table 6, the multiple regression analysis was able to understand the amount of variation explained in the dependent variable “Intention to implement ICT” by the variation in the dependent variables “Technology Management, Data Management, Improved Outcomes, Efficiency, and Software limitation”. Table 6 provides the summary analysis of the regression analysis.

**Table 6:** **Summary of multiple regression analysis**

Variables	Efficiency	Improve outcomes	Data management	Technology management	Software limitation
R values			.724		
R2 value			.524		
Adj R2 value			.498		
F value			20.013		
Sig level			.000		
B value	.257	.349	.030	.044	.092
Beta value	.354	.492	.118	.153	.049
T value	4.523	6.360	1.248	1.583	.669
Sig	.000	.000	.2215	.117	5.505

It can be seen from Table 6 R = .724, with p < .05; this shows that there is a significant relationship between the dependent and independent variables. The adjusted r-square (r = .524) shows that 52.4% of the variation in the independent variable “Intention to implement ICT” is explained collectively by the variation in dependent variables (Technology Management, Data Management, Improved Outcomes, Efficiency, and Software Limitations). Furthermore this was also aligned through the degree of freedom analysis, F- statistics shows that degree of freedom F (5, 91) is 20.0 at a significance level p < .05, indicating the independent variables are significantly related to the dependent variable; hence, the multiple correlation coefficient is significant as well. To further understand the relationship of the individual independent variables on the dependent variable, the “Coefficient Analysis” was studied.

This analysis also shows that independent variables “Efficiency” and “Improved Outcomes” are significant at p < .05 with Beta values of .35 (t = 4.5), and .49 (t = 6.3) respectively, whereas the independent variables “Data management”, “Technology Management” and “Software Limitation” are not significant (t = 0.5, p > .05, t = 1.5, p > .05 and t = 0.6, p > .05) in relation to dependent variable “Intention to implement ICT”. Therefore, the independent variables “Efficiency” and “Improved Outcomes” are major contributors to explaining some of the variance in the dependent variable “Intention to implement ICT” in the chosen healthcare environment.

It can be observed from Table 6 that the variables “Software Limitations” and “Data Management” are not significant and have a negative influence. In order to understand the total impact of the positive influence of the variables Technology Management, Improved Outcomes, and Efficiency, without the variables Data management and Software Limitations, further multiple regressions were conducted by dropping the latter two variables from the analysis.

It was noticed that the ability to explain the variations changed substantially and the variable “Technology Management” impacted significantly on the predication of the dependent variable “Intention to implement ICT”. It was also noticed that the value of “R” (.769) and “R-Square” (.578) improved. These values are summarised in Table 7.

**Table 7:** **Final summary adjusted model for multiple regression analysis**

Variables	Efficiency	Improve outcomes	Technology management
R values		.769	
R2 value		.592	
Adj R2 value		.579	
F value		42.52	
Sig level		.000	
B value	.395	.379	.054
Beta value	.354	.406	.153
T value	4.523	2.373	2.219
Sig	.000	.000	.029

The multiple correlation coefficient R for the three predictors Efficiency, Improved Outcomes, and Technology Management represents the combined correlation of these three predictors with the dependent variable (R = .769). The adjusted R-Square (R2 = .578) clearly indicates that 57.8% of the variations in the dependent variable of intention to implement ICT can be explained by the three main independent variables Efficiency, Improved Outcomes, and Technology Management as combined predictors. Furthermore the F statistic also confirms that the three predictors are significantly related to the dependent variable. Therefore the absolute magnitude of correlations between the predictors in the population is not only greater than zero for our sample, but it is true for the population as well.

## Discussion

The data analysis indicated two clear trends. The first one is that organisations place little concern on “Software Limitations” and “Data Management” as these were not shown to be significant, and have a negative influence. An explanation for this trend may be that organisations have matured in terms of licensing aspects, and that the organisations considered for this study included large organisations and these organisations are familiar with data management aspects. Furthermore, many healthcare organisations collect data on a daily basis and comply with many regulatory requirements in terms of data reporting, and these practices could have contributed to these insignificant results.

On the other hand, Technology Management, Improved Outcomes, and Efficiency have been identified as significant for ICT implementation. In our previous studies, “Efficiency” has been identified as one of the main reasons for implementing ICT in healthcare. Many reports also point to this fact. “Improved Outcomes” is perceived to be a direct benefit of ICT implementation as ICT can provide clinical, customer relations, accounting and nursing benefits. In fact, a 2004 report of health and ageing indicated that consumers expect that ICT will improve outcomes and this is reflected in this study. The qualitative data has also indicated this and there is a consistent view on this aspect.

In terms of technology management, many health practitioners have asserted that ICT implementation, once properly understood and carried out, will result in proper technology management. While there is ample evidence to this claim, what is not clear is the type of technologies. It is not clear whether respondents of this study indicated clinical technology or generic ICT types. We were mainly concentrating on innovative aspects, and the discussion was mainly on emerging type technologies. So, it could be possible that respondents could have implied that emerging technologies would help to manage technology by realising the integration of clinical and other data systems. In fact, in many of our previous health informatics studies conducted in Australia, India and Pakistan, the integration of clinical and consumer data were highlighted as a direct benefit of ICT integration.

**Figure 1:**
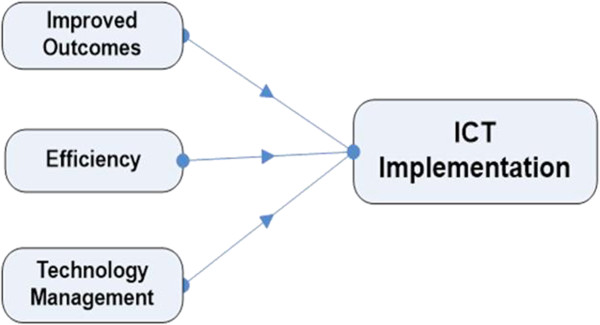
**Initial framework for the factors related to the adoption of wireless technology in HC.**

The three variables, Technology Management, Improved Outcomes, and Efficiency, point to the motivation of ICT implementation and the statistical results point to strong support. These variables are also correlated significantly. The limiting factors such as administrative constraints did not play a crucial role. These indicated the positive sentiments exhibited by the respondents. Thus it is possible to conceive the framework shown in Figure 1.

## Conclusion

This research paper provides some initial findings of the factors that motivate and limit ICT implementation in healthcare organisations. A list of themes which can influence the ICT implementation in a healthcare environment was identified. Some of the themes were already identified in the literature review; however this research also identified new themes, in the form of internal and external factors, which contribute to the general research domain.

### Future research & limitations

In this paper, the initial findings of the first phase of data analysis are presented. Findings are aligned with the previous research. However, the findings of this research will be used to develop further qualitative instruments, a survey questionnaire, in order to test these factors in depth. As indicated earlier, there is a lack of clarity as to the type of technology management and we have decided to investigate these aspects in depth. The research team is already working on this phase of the study. The findings of this research are limited to specific healthcare domains in Australia, India and Pakistan. Further research is still needed to test the findings to generalise the outcomes.

### Ethics statement

To conduct this research a formal approval was sought from the USQ ethic committee and from the respective healthcare facilities as well. Furthermore, a consent form was signed by all participants in the study. It was made clear to all participants that their involvement is voluntary.
